# Official Data and Analytical Forecasts: Differences and Similarities Among Coronavirus Disease (COVID-19) Confirmed Cases and Deaths

**DOI:** 10.3389/fmed.2020.00239

**Published:** 2020-05-19

**Authors:** Ottavia Eleonora Ferraro, Mariangela Valentina Puci, Cristina Montomoli, Sandro Rolesu, Stefano Cappai, Federica Loi

**Affiliations:** ^1^Unit of Biostatistics and Clinical Epidemiology, Department of Public Health, Experimental and Forensic Medicine, University of Pavia, Pavia, Italy; ^2^Osservatorio Epidemiologico Veterinario Regionale della Sardegna, Istituto Zooprofilattico Sperimentale della Sardegna G. Pegreffi, Cagliari, Italy

**Keywords:** COVID-19, pandemic, Italy, public health, standardize mortality rate, official data, risk management

## Introduction

The novel coronavirus disease (COVID-19) is spreading widely with an exponential growth infection rate in several countries worldwide: up to May 5th, 2020, about 3,517,345 cases and 243,401 deaths have been confirmed (1). In Europe overall, about 1.5 million official cases have been reported, and Spain, Italy, and the United Kingdom are the most affected countries. From the 31th of January, in order to better control the virus spread, the Italian government declared an “emergency state,” which is characterized by the implementation of massive containment measures (2, 3).

As now in China, the risk of COVID-19 spreading to other countries is a great concern, as well as the perspective of a secondary cases wave, and given that no vaccine is currently available, rapid and specific diagnostic procedures are an essential tool to allow accurate information of the disease (4, 5). Furthermore, reliable and timely data are fundamental tools to guide the right political and health interventions and to better understand the virus spread.

Since the first Italian spread of the disease from the highest risk area (Northern Italy) to the rest of the nation (the 2nd of March, 2020), the Italian Department of Civil Defense (DCD) have published official reports on COVID-19 distribution to all the Italian regions and provinces (6). The daily regional reports have provided data about number of tests executed (“Tamponi”) and the total COVID-19 cases (“Casi totali”), and details cover recoveries (“Dimessi/Guariti”) and the number of people who have died (“Deceduti”), who are hospitalized with symptoms (“Ricoverati con sintomi”), who are hospitalized in Intensive Care Units (ICUs) (“Terapia intensiva”), and who are in house isolation (“Isolamento domiciliare”). The smallest administrative units in which the data are aggregate are the Italian provinces. All these data are currently used by several scientists, stakeholders, and politicians to understand the daily disease evolution and forecast the possible disease spread in Italy (7–9).

Moreover, it should be noted that the estimates should be critically evaluated to identify their weaknesses and strengths. Data provided in the Italian general reports are based on the province and municipality of patient's residence. This generates a major critical issue on the correct identification of the municipality where virus spread occurred, given that, when applied to COVID-19 cases detected in Assisted Health Residences (AHR), it may generate misleading information. Indeed, given that not all municipality territories includes AHR, several elderly people are hospitalized in commons different from their residence. In these cases, the formal residence of patients has not necessarily changed, and no national regulation about this is available. As a consequence, most of COVID-19 cases that became infected inside the AHR are registered as if they have become infected in the municipality where they were a “resident.” The incorrect attribution of the viral contagion location causes the spread of misleading information on the geographical incidence of the disease as well as the infection, recovery, and lethality estimation rates.

In fact, one of the main important things in epidemiological studies is the ability to define as accurately as possible the reference population, i.e., the rate denominator (tested population). In this context, the number of COVID-19 laboratory tests executed, the sampling method, as well as the number of people tested, is a controversially issue. The choice of people to be tested is regulated by National Law ^1^, but each Region can apply most specific rules. Furthermore, in hospital, the local management can decide on the testing regime used for personnel.

Finally, all this leads to a situation where the number of tests executed does not reflect the number of people tested in specific areas, as the same person may be tested one, two, or three times. Only the declaration of recovery is bound by the performance of at least three tests (one to be declare positive and two to be declare recovered)^1^. Based on this assumption, the number of people tested could be better estimated by subtracting the double number of recoveries from the number of tests executed. However, also this estimation is probably not fully reliable given that healthcare staff could be tested more than once to confirm the negative state of COVID-19, and this would invalidate the data of the reference population.

We must consider the time taken to provide a laboratory result: as reported by many sources, although the maximum time to communicate the swabs result should be 36 h, the results are frequently delayed due to the overload of laboratories. Therefore, the number of positive/recovered cases reported daily could be imprecise and include swabs results related to several days before. In any case, the daily data reported are not comparable, and recovery/lethality/infection rates cannot be properly estimated. Methods proposed by Ghiani et al. recently applied for the Sardinia region, could be more appropriate (10, 11). Furthermore, the declaration of death related to COVID-19 needs to be confirmed by usual and official laboratory tests based on swabs, and these create a well-known problem of the underestimation of deaths. Several estimations for the real numbers of COVID-19 deaths have been provided not only for Italy but also for China, South Korea, and the European Union (12).

## Standardized Mortality Rates Analysis

The Italian National Institute of Statistic (ISTAT) provides official data on the number of deaths for all causes at the municipality level, and they have done so for January, February, March, and first 2 weeks of April of 2015–2020, in a selected number of municipalities (13). These data have been used to evaluate the hypothesis that an increment of the mortality rate could be related to COVID-19 causes.

Data regarding 6,866 municipalities (80% of the total) in 20 Italian regions, divided into 21 age classes (each lasting 5 years) and 3.5 months for a total of 914,622 records, have been recorded by the ISTAT website (13). A total of 247,978 observations have been excluded since 2020 data were not available, and the analysis has been performed based on 4,433 Italian municipalities.

As demonstrated in several previous studies, the standardized mortality rates (SMRs) are generally used to compare the observed event (i.e., mortality in 2020) in the cohort under study with the expected one, which is obtained using the rate of events in a reference population (i.e., mortality during the period between 2015 and 2019) (14).

Collecting data at a regional level, based on resident population at each year in the study (available at: http://demo.istat.it/index.html), mortality rates × 10,000 people (observed mortality rate) have been calculated by age classes by taking into account the observed data from the 1st of January to the 15th of April of each year (2015–2020). By multiplying the median age-mortality rate of the 2015–2019 period by the resident population of 2020 for each age class, the expected age-mortality rate for the 2020 period has been estimated for each Italian region. The SMR, obtained by the ratio between observed and expected 2020 mortality rate, has been calculated for each age class at a regional level, and the same has been done for its 95% Confidence Intervals (95% CI). The exact confidence interval was calculated for each estimated SMR assuming a Poisson process (15).

An SMR > 1 shows an excess of mortality, while an SMR < 1 whos a “shortage” in mortality. If 95% CI includes the null value “1,” which cannot be considered statistically significant, the interpretation is that there is no significant excess/deficit in the mortality rate in the studied population compared to the general population.

**Figure 1:**
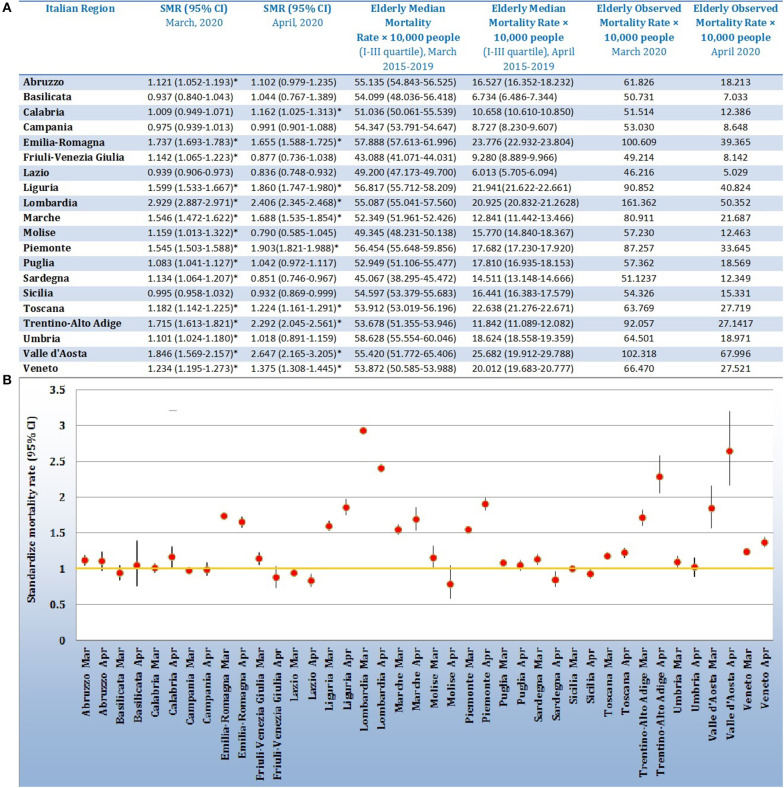
**(A)** Table reporting regional data of Standardize Mortality Rates and 95% Confidence Interval (SMR, 95% CI); median value of mortality rate × 10,000 people in elderly category (75 years or more), related to March and April of 2015–2019; mortality rate × 10,000 people in elderly category (75 years or more) related to March and April of 2020. **(B)** Forest plot reporting the Standardize Mortality Rates values with 95% CI for the 20 Italian Regions for March and April, 2020. The yellow line represents the limit of one, defining the statistical significance (if not included in the 95% CI).

The results, reported in Figure 1, underline an overall excess of deaths in Italy amongst elderly people over 75 years of age in 15 Italian regions in March 2020 compared to previous years, except for Basilicata, Calabria, Campania, Lazio, and Sicilia; January and February did not show statistically significant differences in mortality rates. A total of 10 regions (Calabria, Emilia-Romagna, Liguria, Lombardia, Marche, Piemonte, Toscana, Trentino-Alto Adige, Valle d'Aosta, and Veneto) have confirmed this increment in the first 2 weeks of April of 2020. The largest increase in mortality rate was detected in Lombardia (SMR = 2.929; 95% CI = 2.887–2.971) in March and in Valle d'Aosta (SMR = 2.647; % CI = 2.165–3.205) in April. The lowest excess in mortality rate was recorded in Puglia, which ahd an SMR value equal to 1.083 (95% CI = 1.041–1.127) in March and in Calabria in April (SMR = 1.162; 95% CI = 1.025–1.313).

Given the increasing attention to the consequences of COVID-19 amongst elderly people (10), the observed excess of SMR in this category at national level has been studied more thoroughly in the elderly Italian region (Sardinia). The Sardinian results, obtained by analyzing data from 327 municipalities (20,045 records), illustrate an excess in mortality rates during March 2020 with a statistically significant SMR equal to 1.134 (95% CI 1.064–1.208). In a total of 20 municipalities, a statistically significant SMR > 1 was detected: Assolo, Bottidda, Burcei, Cabras, Calangianus, Galtellì, Gesturi, Monastir, Ossi, Pabillonis, Padru, Porto Torres, Riola Sardo, San Gavino Monreale, Sanluri, Sassari, Sant'Antioco, Siurgus Donigala, Sorgono, and Vallermosa.

Considering the absence of complete national data on COVID-19 related deaths at the municipal level, as well as the lack of information in Italian provincial reports, the issue of death underestimation has been evaluated by comparing SMR results with official COVID-19 cases available for Sardinia (16). Among the 20 municipalities with statistically significant SMR, 11 municipalities (Assolo, Bottidda, Burcei, Galtellì, Gesturi, Monastir, Ossi, Pabillonis, Riola Sardo, Siurgus Donigala, and Sorgono) recorded zero COVID-19 cases during March 2020.

Since public health authorities often need to compare the mortality based on geographical areas, the present work provides a robust overview for those Italian regions with high difference in mortality rates caused by the spread of the pandemic in Italy. The interesting example of excess in mortality, but not officially declared COVID-19 cases, deserves the attention of the health authorities. Furthermore, it must be underlined that the SMRs obtained are underestimated, given that the 2020 mortality rate estimate includes deaths related to incidents in or outside of the work place, or road accidents, which are drastically reduced due to the lockdown period. The inclusion of 80% of the total Italian municipalities has, at least partially, limited the possible bias, providing an important start point for the estimation of real COVID-19 pandemic consequences. However, taking into account the bias of the usage of the 2019 resident population to estimate the 2020 mortality rate, the overall excess of people who have died is about 16,000 deceased, which is in line with the estimates reported in the last ISTAT report (17).

## Discussion

As reported by the World Health Organization in their Pandemic Influenza Risk Management Guidelines (18), influenza pandemics are unpredictable but recurring events, and advance planning and preparedness are critical to help mitigate the impact of a pandemic. Furthermore, taking into account the lessons learned from previous pandemics is a fundamental part of ensuring the adequacy of health strategies in the field.

To date, the health organizations have tried to cope with the emergency; however, a better local health organization is now necessary and can be applied. These problems in official data generate an important issue related to the information provided by the authorities. This is likely not only an Italian issue, but Italy is merely an example for a general need for improved healthcare information collection systems.

The main focus that needs to be taken into account should be the localization of the virus and not only of the infected people. Thus, the correct identification of infected people and their localization is essential for a robust epidemiological analysis and mortality rate estimation. The hypothesized subsequent phase (Italian Phase 2) must necessarily be carried out on the basis of these assumptions. This will allow for, as much as possible, the understanding of the true prevalence of the disease compared to the official cases diagnosed.

The survey should be based on the most relevant characteristics of the population (i.e., sex, age, residence, comorbidities, and symptoms) in order to provide a valid risk analysis and predict the spread of infection. Future investigations could start based on this increase in mortality rate at municipal level and testing the personal contacts of the deceased. On the other hand, simple corrections in data collection and its transmission (i.e., time of sample, localization by residence or hospitalization, and number of swabs/person) could be fundamental tools with which to plan the next steps. This could be applied first in regions with a low population, where additional field sanitary measures will facilitate faster virus localization and promote a spatial epidemiological analysis. Collaboration amongst nations should be encouraged, as that the virus is not bounded by geographical limits.

## Author Contributions

FL performed data collection and *ad hoc* database and supported the statistical analysis on SMR performed by OF. OF, MP, CM, SR, FL, and SC contributed equally in conceptualization, literature search, and writing. and SC and OF took care of the figures. All authors contributed equally in study design, data interpretation, and revision of the manuscript.

## Conflict of Interest

The authors declare that the research was conducted in the absence of any commercial or financial relationships that could be construed as a potential conflict of interest.

## Footnotes

^1^http://www.trovanorme.salute.gov.it/norme/renderNormsanPdf?anno=2020&codLeg=73799&parte=1%20&serie=null
